# Predicting the Suitable Geographical Distribution of *Sinadoxa Corydalifolia* under Different Climate Change Scenarios in the Three-River Region Using the MaxEnt Model

**DOI:** 10.3390/plants9081015

**Published:** 2020-08-11

**Authors:** Xiaotao Huang, Li Ma, Chunbo Chen, Huakun Zhou, Buqing Yao, Zhen Ma

**Affiliations:** 1 Key Laboratory of Restoration Ecology for Cold Regions Laboratory in Qinghai, Northwest Institute of Plateau Biology, Chinese Academy of Sciences, Xining 810008, China; xthuang@nwipb.cas.cn (X.H.); mali@nwipb.cas.cn (L.M.); bqyao@nwipb.cas.cn (B.Y.); mazhen@nwipb.cas.cn (Z.M.); 2 Key Laboratory of Adaptation and Evolution of Plateau Biota, Chinese Academy of Sciences, Xining 810008, China; ccb_8586@ms.xjb.ac.cn; 3 University of Chinese Academy of Sciences, Shijingshan District, Beijing 100049, China; 4 Xinjiang Institute of Ecology and Geography, Chinese Academy of Sciences, Urumqi 830011, China

**Keywords:** suitable distribution, MaxEnt, three-river region, climate change, environmental variable

## Abstract

*Sinadoxa corydalifolia* is a perennial grass with considerable academic value as a rare species owing to habitat destruction and a narrow distribution. However, its distribution remains unclear. In this study, we predicted the distribution of *Sinadoxa corydalifolia* in the three-river region (the source of the Yangtze River, Yellow River, and Lancang River) under the context of climate change using the maximum entropy (MaxEnt) model. Under the current climate scenario, the suitable distribution mainly occurred in Yushu County and Nangqian County. The suitable distribution area of *Sinadoxa corydalifolia* covered 3107 km^2^, accounting for 0.57% of the three-river region. The mean diurnal air temperature range (Bio2), temperature seasonality (Bio4), and mean air temperature of the driest quarter (Bio9) contributed the most to the distribution model for *Sinadoxa corydalifolia*, with a cumulative contribution of 81.4%. The highest suitability occurred when air temperature seasonality (Bio4) ranged from 6500 to 6900. The highest suitable mean air temperature of the driest quarter ranged from −5 to 0 °C. The highest suitable mean diurnal temperature (Bio2) ranged from 8.9 to 9.7 °C. In future (2041–2060) scenarios, the suitable distribution areas of *Sinadoxa corydalifolia* from high to low are as follows: representative concentration pathway (RCP)26 (6171 km^2^) > RCP45 (6017 km^2^) > RCP80 (4238 km^2^) > RCP60 (2505 km^2^). In future (2061–2080) scenarios, the suitable distribution areas of *Sinadoxa corydalifolia* from high to low are as follows: RCP26 (18,299 km^2^) > RCP60 (11,977 km^2^) > RCP45 (10,354 km^2^) > RCP80 (7539 km^2^). In general, the suitable distribution will increase in the future. The distribution area of *Sinadoxa corydalifolia* will generally be larger under low CO_2_ concentrations than under high CO_2_ concentrations. This study will facilitate the development of appropriate conservation measures for *Sinadoxa corydalifolia* in the three-river region.

## 1. Introduction

The geographical distribution of species, which is an important field of biogeography research, mainly reveals the spatial distribution of species and its relationship with environmental factors [1,2,3]. Climatic and environmental factors (terrain and soil, among others) are the decisive factors affecting species distribution [3,4,5]. Global warming is expected to continue, with the average temperature of the earth increasing by 0.3–4.5 °C by 2100 compared with the average temperature from 1986 to 2005. Climate change has caused substantial changes in the geographical distribution of many species [2,6,7]. Understanding the geographical distribution of species is an important basis for the delimitation of protected areas, the control of invasive species, and the conservation of biodiversity [8,9]. At present, predictions of the impact of climate change on species distribution have become a popular and key field of biogeography and global change biology.

With the improvement of research methods and computing ability and the enrichment of data acquisition, there is great potential for studying the suitable distributions of species and their association with abiotic factors using species distribution models in combination with climate and environmental data [10,11,12]. We can use these models to determine species distributions, limiting factors and habitat conditions. Various species distribution models have been developed to evaluate the ecological responses and distribution areas, such as CLIMEX, Domain, genetic algorithm for rule set production (GARP), and maximum entropy (MaxEnt). Previous studies showed that MaxEnt performed better than other models when the sample sizes were small [13,14,15]. Thus, MaxEnt has become increasingly widely used.

*Sinadoxa corydalifolia* is a perennial grass with considerable academic value as it is a rare species in the three-river region of the Qinghai–Tibet Plateau owing to habitat destruction and a narrow distribution. It is an important genetic resource and was discovered as a new species in 1964 [16,17]. This species grows in gravel belts and over moist ground in canyons at altitudes of 3900 m to 4800 m above sea level. This species has been found in only Yushu County and Nangqian County, which are in the three-river region of Qinghai Province, China [16]. If reasonable protection is not implemented for this species, it will be in danger of extinction. Previous studies showed that the formation of this species was closely related to the special natural environment (climate changes and complex terrain, among others) caused by the uplift of the Qinghai Tibet Plateau [16,17]. However, at present, no study has reported on the suitable distribution of *Sinadoxa corydalifolia* and its correlation with environmental factors in the three-river region, which is very detrimental to the scientific protection of the species [18,19,20].

The three-river region is located in the hinterland of the Qinghai–Tibet Plateau. Because alpine plants in this region are highly sensitive to abnormal surface climates, their evolution is often associated with surface climate changes, life history, and human origin and evolution. In addition, the three-river region has served as an ideal laboratory for research in ecology, geology, and evolutionary biology because of its unique geology and geographical environment [21,22,23]. Therefore, the study of the species distribution in the three-river region and its relationship with environmental factors is helpful to understand the processes and mechanisms of biological adaptation and diffusion under complex climatic and environmental conditions, and the results of such a study would provide a theoretical basis and supporting data for biodiversity conservation and agricultural production management in this region.

In this study, we predict the distribution of *Sinadoxa corydalifolia* in the three-river region using the MaxEnt model. The objectives of the present study include (1) identifying a suitable habitat for *Sinadoxa corydalifolia* under the current climate scenario and the most important environmental factors affecting the range of *Sinadoxa corydalifolia* and (2) examining a suitable habitat for *Sinadoxa corydalifolia* under future climate scenarios in the three-river region.

## 2. Materials and Methods

### 2.1. Study Area

**Figure 1:**
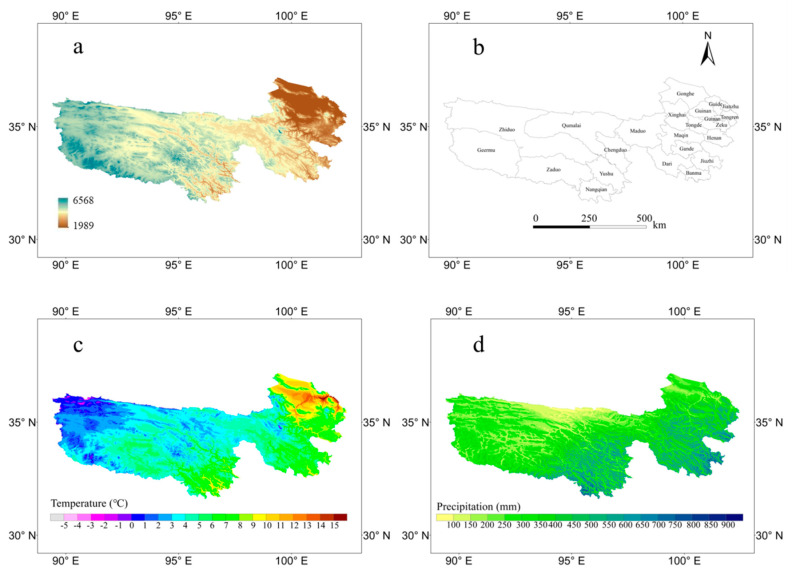
Distributions of the elevation (**a**), administrative division (**b**), mean temperature of the growing season (**c**), and total precipitation of the growing season in the three-river region (**d**).

The three-river region, which is the source of the Yangtze River, the Yellow River, and the Lancang River, is located in the hinterland of the Qinghai–Tibet Plateau. This region has the highest concentration of biodiversity in the alpine region and has the reputation of a “plateau species gene pool” [9,24]. The climate in this region is a typical plateau continental climate, mainly with alteration of cold and hot seasons and dry and wet seasons [25,26] (Figure 1). Plants in this region are extremely sensitive to climate change and human disturbances because of the special geographical location. Climate change and unreasonable human activities have led to serious damage to species resources in this region [27,28,29].

### 2.2. Data

**Figure 2:**
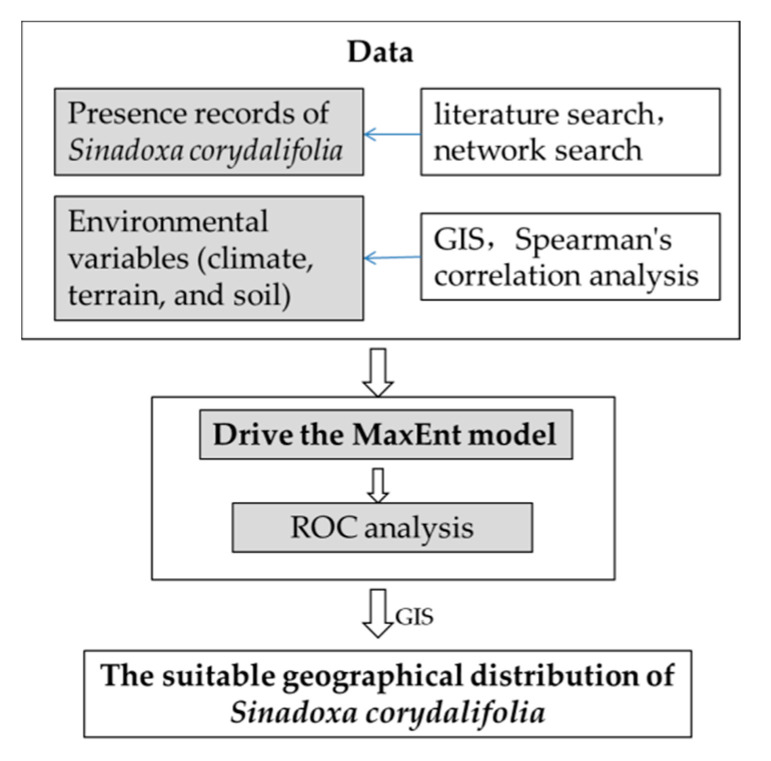
Research chart. GIS, geographic information system; ROC, receiver operating characteristic; MaxEnt, maximum entropy.

Distribution point data of *Sinadoxa corydalifolia* and environmental variable data were needed to drive the MaxEnt model to simulate and validate the distribution of the species (Figure 2).

#### 2.2.1. Presence Records of *Sinadoxa Corydalifolia*

We conducted an extensive literature search and referred to the Global Biodiversity Information Facility (GBIF, https://www.gbif.org/) and the Chinese Virtual Herbarium databases (CVH, http://v5.cvh.org.cn/), among others. When occurrence records lacked exact geo-coordinates, we used Google Earth (http://ditu.google.cn/) to determine the latitude and longitude according to the described geographical locations. A total of eight documented records of *Sinadoxa corydalifolia* presence in the three-river region were obtained for constructing the models. In accordance with the requirements of the MaxEnt model, the presence records of the target species were organized into “.csv” format files.

#### 2.2.2. Environmental Variables and Processing

Environmental variables are the key factors affecting species distribution. We initially selected 25 environmental factors that might influence the distribution of *Sinadoxa corydalifolia*. These factors included 21 bioclimatic variables, 3 terrain variables, and soil texture variables. All the environmental variable data were converted to ASCII format with a 1 km × 1 km spatial resolution using ArcGIS 10.0 software.

The bioclimatic variables were downloaded from the WorldClim-Global Climate Database (http://worldclim.org/) with a 1 km × 1 km spatial resolution. These bioclimatic variables were developed for species distribution modeling and related ecological applications. The variables represent annual trends (e.g., mean annual temperature and annual precipitation), seasonality (e.g., annual range in temperature and precipitation), and extreme or limiting environmental factors (e.g., temperature of the coldest and warmest month and precipitation of the wet and dry quarters). These variables were applied as a baseline for current and future climate scenarios. The bioclimatic variables spanning 1979 to 2013 represent the current climate scenario. The bioclimatic variables representing the future climate scenarios were divided into two periods: the years from 2041 to 2060 and the years from 2061 to 2080. In the 5th report of the IPCC, four representative concentration pathways (RCPs) were established for future climate scenarios, including RCP26 (minimum greenhouse gas emission scenario), RCP45 (medium greenhouse gas emission scenario), RCP60 (high greenhouse gas emission scenario), and RCP85 (highest greenhouse gas emission scenario) [30].

Terrain data included elevation, slope, and aspect. The elevation data with 90 m × 90 m spatial resolution were downloaded from the Shuttle Radar Topography Mission (SRTM) data set of the National Geospatial Data Cloud (http://www.gscloud.cn/). Slope and aspect data were obtained according to surface analysis of elevation data using ArcGIS 10.0 software (Environmental Systems Research Institute, Redlands, CA, USA), and all terrain data were resampled to 1 km × 1 km spatial resolution using ArcGIS 10.0 software.

Soil texture data with a 1 km × 1 km spatial resolution were derived from the Harmonized World Soil Database (HWSD) based on a recent 1:1,000,000 scale soil map of China (http://westdc.westgis.ac.cn/data/611f7d50-b419-4d14-b4dd-4a944b141175), which was derived from the second national land survey.

Some environmental variables might be spatially correlated, resulting in inaccurate simulation results [13,31,32]. To eliminate the influence of collinearity on the modeling process and interpretation of the results, we used Spearman’s correlation analysis to examine the cross-correlation and removed variables with low contributions when the correlation coefficient between environmental variables was greater than or equal to 0.8. Out of 25 variables, only 12 were selected as evaluator variables: mean diurnal air temperature range (Bio2), isothermality (Bio3), air temperature seasonality (Bio4), mean air temperature of the wettest quarter (Bio8), mean air temperature of the driest quarter (Bio9), precipitation of the wettest month (Bio13), precipitation of the driest month (Bio14), precipitation seasonality (Bio15), precipitation of the coldest quarter (Bio19), elevation, slope, and aspect (Table 1).

### 2.3. MaxEnt Model

A species distribution model mainly uses georeferenced occurrence records of species and the related environmental data to estimate the ecological position of species, and these positions are projected onto the landscape according to the specific algorithm. The modeling results can be explained as the probability of species occurrence, habitat suitability, or species richness [33,34,35]. The geographical distribution of species and species diversity and richness depend on whether people understand their niches. A niche is a sampling feature of species in the internal position of the community. The niche of a species depends on the spatial and temporal distribution of resource gradients that contribute to species evolution [12,36,37]. The measurement of environmental factors that quantify the distribution and richness of species is an important step in strengthening the understanding of species niches. In general, most species distribution models based on niches are developed by the study of plant, animal, and biodiversity. Among these models, MaxEnt is one of the most widely used thanks to its good prediction accuracy. This model can analyze species distribution by integrating georeferenced occurrence records and related environmental data and express the suitability of areas for species under different environmental variables through raster layers. MaxEnt has the advantages of simple operation, fast operation speed, and good prediction results. MaxEnt can perform better than other models when faced with small sample sizes [13,38,39,40].

MaxEnt Version 3.4.1 (Columbia University, New York, NY, USA) (http://www.cs.Princeton.edu/~schapire/MaxEnt) was used in this study. We selected a random seed and set the regularization multiplier to 1.1, random test percentage to 25, and replicates to 4. Next, we imported presence records of *Sinadoxa corydalifolia* and environmental data into the model and selected the options for drawing response curves and jackknife sampling. The other settings were left at the default options. Then, the model was run to predict the distribution of *Sinadoxa corydalifolia*. We converted the ASCII files (output files) to grid files to obtain the spatial distribution map of *Sinadoxa corydalifolia* in the three-river region using ArcGIS 10.0 software.

The results of the modeled species distribution are continuous values that vary between 0 and 1. High values represent a high degree of species adaptation, which means that the species is likely to exist in this region. In contrast, the low values indicate the opposite. The habitat of *Sinadoxa corydalifolia* was divided into four types: not suitable (value ≤ 0.3), generally suitable (0.3 < value ≤ 0.5), moderately suitable (0.5 < value ≤ 0.7), and highly suitable (0.7 ≤ value).

In this study, a set of georeferenced records of *Sinadoxa corydalifolia* occurrence were divided into two parts: 75% of the data were used to drive the model, and 25% of the data were used to validate the modeling results. To calibrate and validate the robustness of the evaluation for the MaxEnt model, threshold-independent receiver operating characteristic (ROC) analysis was used. The area under the ROC curve (AUC) was examined for additional precision analysis. The AUC can be obtained by calculating the area below the ROC curve. The value of AUC ranges between 0.5 and 1. A high AUC indicates more accurate results. Modeling results are considered to have high accuracy when AUC > 0.9. In general, AUC > 0.85 means the modeling results can be adopted (Figure 2).

## 3. Results

### 3.1. Model Validation

**Figure 3:**
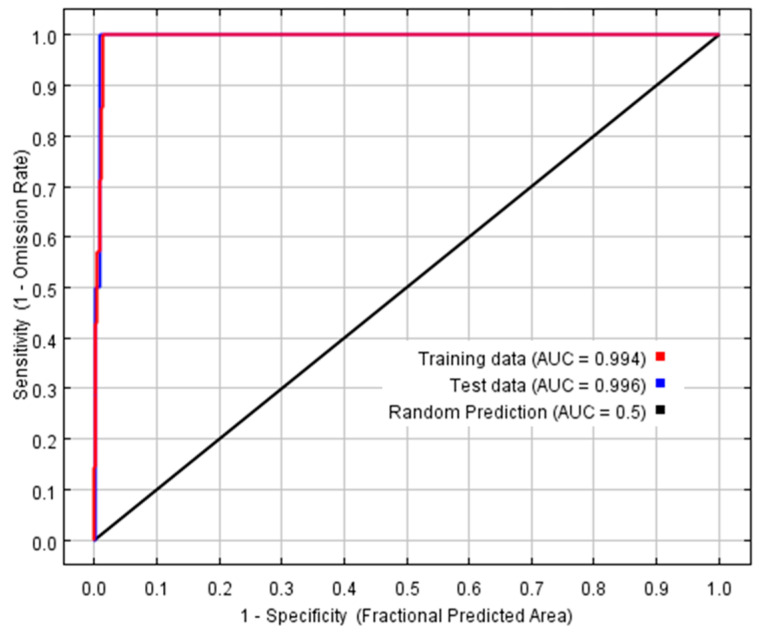
Receiver operating characteristic curve with the area under the curve (AUC).

Validation guarantees the reliability of modeling results. In this study, the AUC value showed that the MaxEnt model performed well (AUC = 0.994) under the current scenario (Figure 3).

### 3.2. Predicted Current Potentially Suitable Distribution

**Figure 4:**
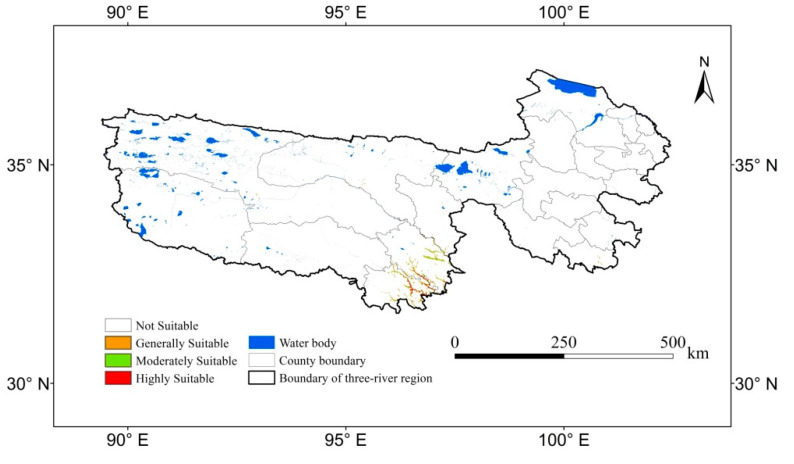
The current potentially suitable distribution for *Sinadoxa corydalifolia.*

Under the current climate scenario, the suitable distribution mainly occurred in Yushu County and Nangqian County. The suitable distribution area of *Sinadoxa corydalifolia* was 3107 km^2^, accounting for 0.57% of the three-river region. The high, medium, and low suitable areas covered 726 km^2^, 972 km^2^, and 1409 km^2^, respectively, representing 0.13%, 0.18%, and 0.26% of the three-river region, respectively (Figure 4).

**Figure 5:**
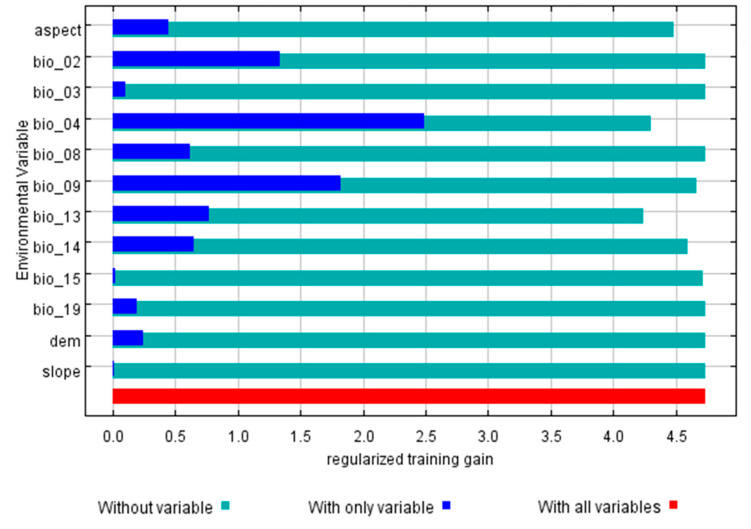
Jackknife plot for training gain.

MaxEnt’s jackknife test tool can evaluate the impact of each environmental variable on the prediction of the *Sinadoxa corydalifolia* distribution. The MaxEnt model automatically outputs the contribution rate of each variable [41,42]. The top three environmental factors were the mean diurnal air temperature range (Bio2), temperature seasonality (Bio4), and mean air temperature of the driest quarter (Bio9) (Figure 5). The contributions from high to low are as follows: temperature seasonality (Bio4) (34.4%), mean air temperature of the driest quarter (Bio9) (27.6%), and mean diurnal air temperature range (Bio2) (19.4%). The cumulative contributions of these three factors reached values as high as 81.4%, indicating that these three factors were the key factors affecting the distribution and adaptability of *Sinadoxa corydalifolia* (Table 2).

**Figure 6:**
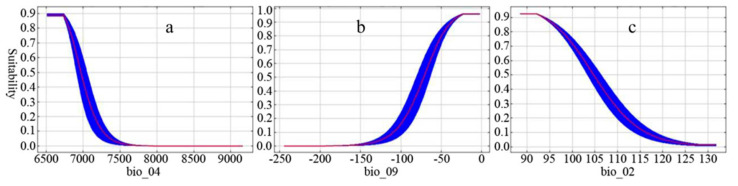
Response curves of *Sinadoxa corydalifolia* to air temperature seasonality (bio_04) (**a**), mean air temperature of the driest quarter (bio_09) (**b**) and mean diurnal temperature (bio_02) (**c**).

Figure 6 shows the response curves of *Sinadoxa corydalifolia* to the three variables with the greatest contributions to habitat suitability. A suitability value greater than 0.5 indicates that the interval is suitable for the growth of *Sinadoxa corydalifolia*. Figure 6a shows that the interval of suitable air temperature seasonality ranged from 6550 to 7000. The highest suitability occurred when air temperature seasonality ranged from 6550 to 6750, indicating that *Sinadoxa corydalifolia* has a small temperature tolerance range. Figure 6b shows that the interval of the suitable mean air temperature of the driest quarter ranged from −7.2 to −0.4 °C. The highest suitable mean air temperature of the driest quarter ranged from −2.5 to −0.4 °C. Figure 6c shows that the interval of the suitable mean diurnal temperature ranged from 8.8 to 10.5 °C. The highest suitable mean diurnal temperature ranged from 8.8 to 9.2 °C.

### 3.3. Predicted Future Suitable Distribution

**Figure 7:**
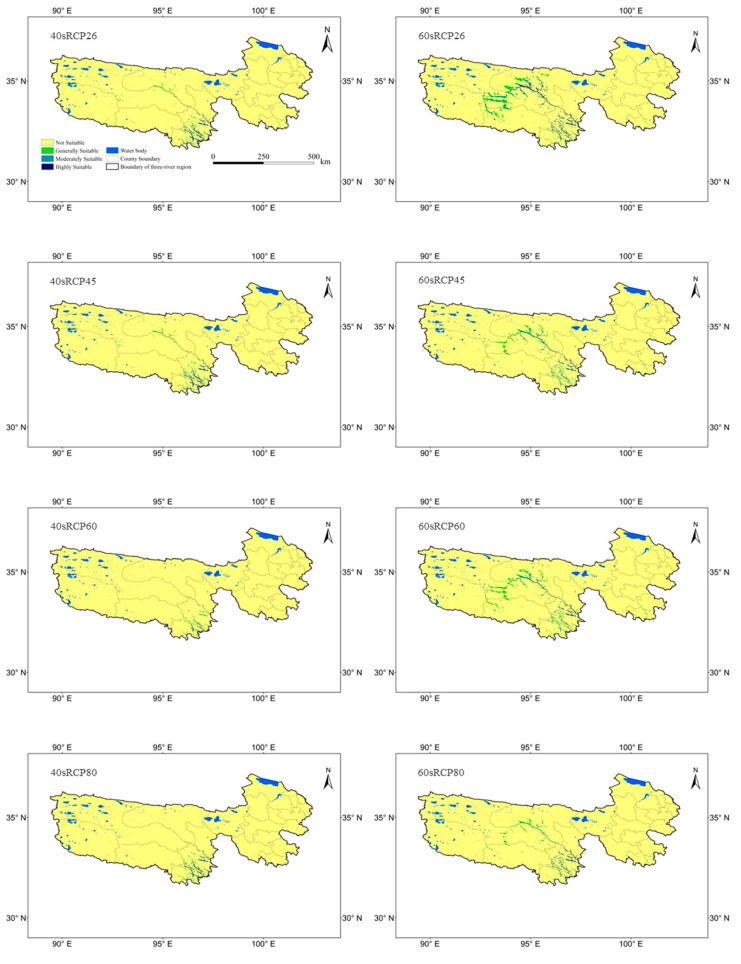
The suitable distribution of *Sinadoxa corydalifolia* under future scenarios (note: 40sRCP26 represents the minimum greenhouse gas emission scenario from 2041 to 2060, 40sRCP45 represents the medium greenhouse gas emission scenario from 2041 to 2060, 40sRCP60 represents the high greenhouse gas emission scenario from 2041 to 2060, 40sRCP80 represents the highest greenhouse gas emission scenario from 2061 to 2080, 60sRCP26 represents the minimum greenhouse gas emission scenario from 2061 to 2080, 60sRCP45 represents the medium greenhouse gas emission scenario from 2061 to 2080, 60sRCP60 represents the high greenhouse gas emission scenario from 2061 to 2080, and 60sRCP80 represents the highest greenhouse gas emission scenario from 2061 to 2080). RCP, representative concentration pathway.

In the RCP26 scenario from 2041 to 2060, the suitable distribution area was 6171 km^2^, accounting for 1.12% of the three-river region. Compared with the current distribution, the suitable distribution in Yushu County, Nangqian County, and Qumalai County obviously increased (Figure 7).

In the RCP45 scenario from 2041 to 2060, the suitable distribution area covered 6017 km^2^, accounting for 1.10% of the three-river region. Compared with the current distribution, the suitable distribution in Yushu County, Nangqian County, Qumalai County, Zhiduo County, and Chengduo County obviously increased (Figure 7).

In the RCP60 scenario from 2041 to 2060, the suitable distribution mainly occurred in Yushu County and Nangqian County. The suitable distribution area covered 2505 km^2^, accounting for 0.46% of the three-river region. Compared with the current distribution, the suitable distribution area obviously decreased (Figure 7).

In the RCP80 scenario from 2041 to 2060, the suitable distribution mainly occurred in Yushu County and Nangqian County. The suitable distribution area covered 4238 km^2^, accounting for 0.77% of the three-river region. Compared with the current distribution, the suitable distribution area obviously increased (Figure 7).

In the RCP26 scenario from 2061 to 2080, the suitable distribution mainly occurred in Yushu County, Nangqian County, Qumalai County, Zhiduo County, and Zaduo County. The suitable distribution area covered 18,299 km^2^, accounting for 3.33% of the three-river region. Compared with the future distribution under the 40sRCP26 scenario, the suitable distribution area obviously increased (Figure 7).

In the RCP45 scenario from 2061 to 2080, the suitable distribution mainly occurred in Yushu County, Nangqian County, Qumalai County, Zhiduo County, and Zaduo County. The suitable distribution area covered 10,354 km^2^, accounting for 1.89% of the three-river region. Compared with the distribution under the 40sRCP45 future scenario, the suitable distribution area in the 60sRCP45 future scenario obviously increased (Figure 7).

In the RCP60 scenario from 2061 to 2080, the suitable distribution mainly occurred in Yushu County, Nangqian County, Qumalai County, Zhiduo County, and Zaduo County. The suitable distribution area covered 11,977 km^2^, accounting for 2.18% of the three-river region. Compared with the distribution in the 40sRCP60 future scenario, the suitable distribution area obviously increased (Figure 7).

In the RCP80 scenario from 2061 to 2080, the suitable distribution mainly occurred in Yushu County, Nangqian County, Qumalai County, Zhiduo County, and Zaduo County. The suitable distribution area covered 7539 km^2^, accounting for 1.37% of the three-river region. Compared with the distribution in the 40sRCP80 future scenario, the suitable distribution area in the 60sRCP80 future scenario obviously increased (Figure 7).

In general, the suitable distribution area increased in the future. The suitable distribution mainly extended to the northwest. Under different CO_2_ concentration scenarios, the suitable distribution areas from 2041 to 2060 from large to small were as follows: RCP26 > RCP45 > RCP80 > RCP60, and the suitable distribution areas from 2061 to 2080 from large to small were as follows: RCP26 > RCP60 > RCP45 > RCP80 (Table 3).

## 4. Discussion

### 4.1. Uncertainty of the Results

Previous studies showed that the MaxEnt model performed better than other models when the sample sizes were small [13,14,15]. In addition, the simulation results of the MaxEnt model were validated by the AUC value, which indicated that the accuracy was high. However, owing to the complexity of the real world, there were still some uncertainties in the simulation results, as in any other model. The uncertainty was mainly caused by two factors, including input data and the model structure itself [1,43].

Only eight sample points were considered owing to the sparse distribution of *Sinadoxa corydalifolia*, which inevitably affected the simulation accuracy. The longitude and latitude information of some sample points was not clear enough and needed to be confirmed through Google Earth, resulting in a certain degree of uncertainty in the results. The data collection ages of these sample points were not uniform and exhibited a large span. In some sampling points, the original species might have disappeared in the decades after sampling because the environmental variables required for this species also exhibited a certain degree of variation, which might conceal the distribution information existing in the sample collection points. These environmental variables and species information used for simulation directly led to the decline in the accuracy of the simulated species distribution [37,43].

In the MaxEnt model, parameter adjustment and results’ validation were conducted at the same time. Sample point data were used alternately for parameter adjustment and validation. Thus, this method inevitably had a negative impact on the accuracy of the results [32,42,44].

### 4.2. The Relationship between the Distribution and Environmental Factors

Previous studies showed that *Sinadoxa corydalifolia* has high requirements for its growth environment [9,16]. In this study, according to the contributions of the evaluation factors, the mean diurnal air temperature range (Bio2), temperature seasonality (Bio4), and mean air temperature of the driest quarter (Bio9) made the greatest contributions to the distribution of *Sinadoxa corydalifolia*, and the cumulative contribution was as high as 81.4%, which suggested that temperature was the decisive factor affecting the regional distribution of *Sinadoxa corydalifolia*. This result was consistent with the fact that *Sinadoxa corydalifolia* was found in only the Yushu area, which is a typical alpine region [9,16]. Bio13 (precipitation of the wettest month) and Bio14 (precipitation of the driest month) are related to precipitation. The sum of the contributions of these two factors was 11.4%, which suggested that precipitation was also an important factor affecting the distribution of *Sinadoxa corydalifolia*. This result is similar to that in the study by Wang et al. [9,16]. The contribution of aspect to the distribution of *Sinadoxa corydalifolia* was 3.7%, which suggested that aspect also had a certain influence on the distribution of *Sinadoxa corydalifolia*. This result was supported by the fact that *Sinadoxa corydalifolia* was found in canyons or deep ditches [9,16]. In general, our simulation results are in line with those in a previous publication by Wang et al. [16], which suggested that *Sinadoxa corydalifolia* grows in gravel belts and moist ground in canyons at altitudes of 3900 m to 4800 m above sea level.

### 4.3. Change in the Spatial Distribution under Climate Change

There are several reports on the prediction of suitable species distributions under climate change using the MaxEnt model. These reports mainly focus on energy plants, medicinal plants, invasive species, endangered species, and the migration routes of species [4,38,39,42,45,46,47,48]. Different plants have different requirements for their growth environments, resulting in obvious differences in suitable distributions. In this study, a suitable distribution of *Sinadoxa corydalifolia* was predicted for the first time using the MaxEnt model in combination with geographic information systems. According to the prediction, the suitable distribution of *Sinadoxa corydalifolia* in the three-river region is very narrow, indicating that the growth of *Sinadoxa corydalifolia* has high environmental requirements, which is in line with a previous publication by Wang et al. [16]. In our study, the suitable distributions were concentrated in Yushu County and Nangqian County, which are generally consistent with those in previous field records [9,16], indicating that the simulation results in our study are scientific and accurate. With climate change, the suitable distribution was predicted to gradually expand in the future, indicating that the climate in the future would become increasingly suitable for the growth of *Sinadoxa corydalifolia*, particularly in Qumalai County and Zhiduo County, which would provide areas for introducing *Sinadoxa corydalifolia* in the future. The suitable distribution areas differed under different CO_2_ concentration scenarios, indicating that climate change imposes certain uncertainty regarding the suitable distribution of *Sinadoxa corydalifolia*. In general, the distribution area of *Sinadoxa corydalifolia* is larger under relatively low CO_2_ concentrations than under relatively high CO_2_ concentrations, which suggests that the conditions for *Sinadoxa corydalifolia* growth are most suitable in environments with thin air on the plateau [49].

### 4.4. Implications

Previous studies regarding *Sinadoxa corydalifolia* focused on genetic analyses and anatomical characteristics [16,17]. This study is the first systematic investigation of the suitable geographical distribution of *Sinadoxa corydalifolia* under different climate change scenarios in the three-river region. At present, *Sinadoxa corydalifolia* is a valuable species in danger of extinction owing to habitat destruction and its narrow distribution. Our study provides important data for reasonable protection and development of resources. Under the climate change scenario, species will move to areas similar to their current living environments [50]. Thus, adverse human interference should be banned to ensure that *Sinadoxa corydalifolia* will be able to migrate to suitable areas in the future. In addition, we recommend that land management agencies further monitor the current status of the species and then develop a more reasonable management strategy for conservation [18,19,20,51]. *Sinadoxa corydalifolia* is an important genetic resource for species improvement [16], and plants with a narrow distribution usually have constrained ecological adaptability and are more susceptible to the impact of climate change than broadly distributed species [52,53]. Thus, we also recommend that funds should be actively provided to further study this species.

## 5. Conclusions

In this study, we predicted the distribution of *Sinadoxa corydalifolia* in the three-river region in the context of climate change using the MaxEnt model. Under the current climate scenario, the suitable distribution mainly occurred in Yushu County and Nangqian County, which is generally consistent with previous field records. The suitable distribution area of *Sinadoxa corydalifolia* covered 3107 km^2^, accounting for 0.57% of the three-river region. Temperature was the most important factor affecting the regional distribution of *Sinadoxa corydalifolia*. With climate change, the suitable distribution was predicted to be mainly extended to the northwest. The suitable distribution was predicted to gradually expand in the future, indicating that the climate in the future may become increasingly suitable for the growth of *Sinadoxa corydalifolia*, particularly in Qumalai County and Zhiduo County. The distribution area of *Sinadoxa corydalifolia* would be generally larger under low CO_2_ concentrations than under relatively high CO_2_ concentrations, which suggested that the conditions for *Sinadoxa corydalifolia* growth are most suitable in environments with thin air on the plateau. This study will facilitate the development of appropriate conservation measures for *Sinadoxa corydalifolia* in the three-river region.

## Figures and Tables

**Table 1:** Environmental variables selected to drive the maximum entropy (MaxEnt) model.

Code	Variables	Unit
Bio2	Mean diurnal air temperature range (mean of monthly (maximum air temperature–minimum air temperature))	°C * 10
Bio3	Isothermality	Dimensionless
Bio4	Air temperature seasonality	Dimensionless
Bio8	Mean air temperature of the wettest quarter	°C * 10
Bio9	Mean air temperature of the driest quarter	°C * 10
Bio13	Precipitation of the wettest month	mm/month
Bio14	Precipitation of the driest month	mm/month
Bio15	Precipitation seasonality (coefficient of variation)	Dimensionless
Bio19	Precipitation of the coldest quarter	mm/quarter
Elevation	Elevation	m
Slope	Slope	%
Aspect	Aspect	Degree

**Table 2:** Relative contributions of the environmental variables to the maximum entropy (MaxEnt) model.

Variable	Percent Contribution (%)
bio_04	34.4
bio_09	27.6
bio_02	19.4
aspect	7.9
bio_14	4.6
bio_13	3
bio_15	1.2
bio_19	0.9
slope	0.4
bio_03	0.3
bio_08	0.2
dem	0.1

**Table 3:** Suitable distribution areas for *Sinadoxa corydalifolia* (note: 40sRCP26 represents the minimum greenhouse gas emission scenario from 2041 to 2060, 40sRCP45 represents the medium greenhouse gas emission scenario from 2041 to 2060, 40sRCP60 represents the high greenhouse gas emission scenario from 2041 to 2060, 40sRCP80 represents the highest greenhouse gas emission scenario from 2061 to 2080, 60sRCP26 represents the minimum greenhouse gas emission scenario from 2061 to 2080, 60sRCP45 represents the medium greenhouse gas emission scenario from 2061 to 2080, 60sRCP60 represents the high greenhouse gas emission scenario from 2061 to 2080, and 60sRCP80 represents the highest greenhouse gas emission scenario from 2061 to 2080). RCP, representative concentration pathway.

Scenarios		Generally Suitable	Moderately Suitable	Highly Suitable	Perfectly Suitable
current	area (km^2^)	1409	972	726	3107
	Percentage of area (%)	0.26	0.18	0.13	0.57
40sRCP26	area (km^2^)	2505	1595	2071	6171
	Percentage of area (%)	0.46	0.29	0.38	1.12
40sRCP45	area (km^2^)	2378	1538	2101	6017
	Percentage of area (%)	0.43	0.28	0.38	1.10
40sRCP60	area (km^2^)	1173	867	465	2505
	Percentage of area (%)	0. 21	0.16	0.08	0.46
40sRCP80	area (km^2^)	1331	1378	1529	4238
	Percentage of area (%)	0.24	0.25	0.28	0.77
60sRCP26	area (km^2^)	7656	5511	5132	18,299
	Percentage of area (%)	1.39	1.00	0.93	3.33
60sRCP45	area (km^2^)	4942	3259	2153	10,354
	Percentage of area (%)	0.90	0.59	0.39	1.89
60sRCP60	area (km^2^)	6066	3607	2304	11,977
	Percentage of area (%)	1.10	0.66	0.42	2.18
60sRCP80	area (km^2^)	3284	2300	1955	7539
	Percentage of area (%)	0.60	0.42	0.36	1.37
